# Supplementary material for: Causal interplay between lactose intolerance and gut microbiota: a combined bidirectional Mendelian randomization and in vivo validation study
Source: Front Nutr. 2026 Jun 1;13:1803337. doi: 10.3389/fnut.2026.1803337 (PMC13265576; doi:10.3389/fnut.2026.1803337)
Supplement: Supplementary file 1 [file Data_Sheet_1.zip › supplementary materials/Forward/leave one out cross validation/ebi-a-GCST90027462.finngen_R12_E4_LACTONAS.pdf]

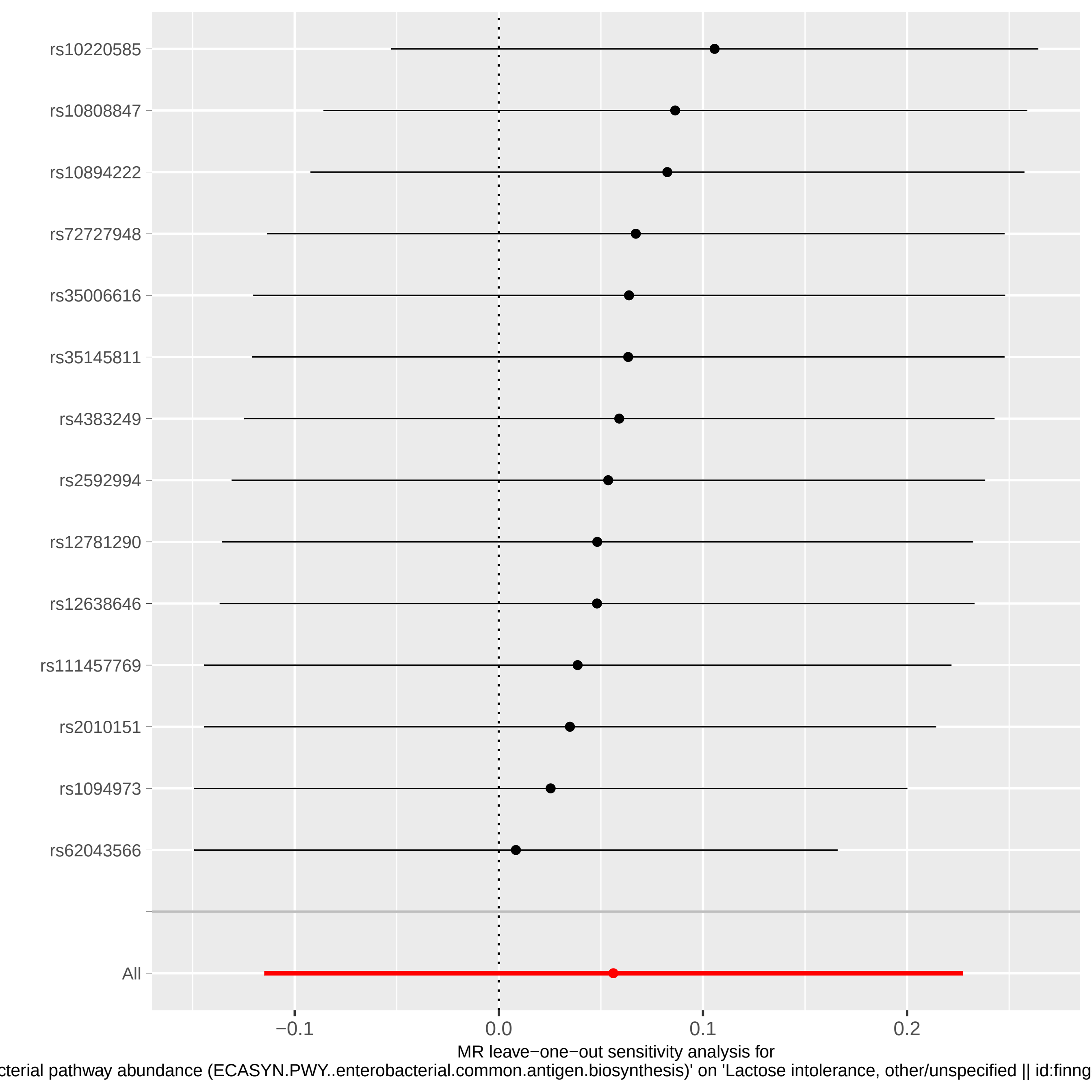
rs10220585
rs10808847
rs10894222
rs72727948
rs35006616
rs35145811
rs4383249
rs2592994
rs12781290
rs12638646
rs111457769
rs2010151
rs1094973
rs62043566
All
−0.1
0.0
0.1
0.2
MR leave−one−out sensitivity analysis for
cterial pathway abundance (ECASYN.PWY..enterobacterial.common.antigen.biosynthesis)' on 'Lactose intolerance, other/unspecified || id:finng
